# Effect of Lambdacyhalothrin on Locomotor Activity, Memory, Selected Biochemical Parameters, Tumor Necrosis Factor α, and Interleukin 1ß in a Mouse Model

**DOI:** 10.3390/ijerph17249240

**Published:** 2020-12-10

**Authors:** Barbara Nieradko-Iwanicka, Michał Konopelko

**Affiliations:** 1 Chair and Department of Hygiene, Medical University of Lublin, Aleje Racławickie 1, 20-059 Lublin, Poland; 2 Department of Otolaryngology and Laryngological Oncology, Independent Public Clinical Hospital No. 4 in Lublin, 20-059 Lublin, Poland; mm.konopelko@gmail.com

**Keywords:** pyrethroids, lambdacyhalothrin, nephrotoxicity, hepatotoxicity, immunotoxicity

## Abstract

Background: Pyrethroids are synthetic insecticides used for plant protection. They are synthetic analogues of pyrethrins. Lambdacyhalothrin (LCH) is a type II pyrethroid used for wheat, potato, corn farming, and malaria control. There are data that pyrethroids may cause neurotoxicity, nephrotoxicity, hepatotoxicity, and immunotoxicity in non-target organisms. Methods: The experiment was carried on 32 Albino Swiss mice (16 females and 16 males). The animals were divided into four groups. Controls received canola oil; the rest received LCH orally in oil at a dose of 2 mg/kg bw for 7 days. Memory retention was assessed in a passive avoidance task on day 2 and 7, and spatial memory and motor activity in a Y-maze on day 1 and 7. Blood morphology, biochemical tests, tumor necrosis factor α, and interleukin 1ß were measured. Results: Decreased white blood cell count and red blood cell count, increased creatinine, and increased kidney and liver mass were observed in groups exposed to LCH. In LCH-exposed males’ kidneys and livers, interleukin 1ß was significantly elevated, and it was correlated with creatinine concentration. Conclusions: Subacute poisoning with a low dose of LCH does not significantly affect memory nor locomotor activity but increases proinflammatory interleukin 1ß in male livers and kidneys and reduces white and red blood cell counts.

## 1. Introduction

Pyrethroids are synthetic insecticides used primarily as plant protection products. They are synthetic analogues of natural pyrethrins. Pyrethrins were obtained from the dried flower baskets of the Dalmatian pyrethrum *Chrysanthemum Cinerariaefolium Vis*. From the extraction of the flower of *Ch. Cinerariaefolium* resulted in six insecticides: pyrethrin I, pyrethrin II, jasmoline I, jasmoline II, cinerin I, and cinerin II [1,2]. We divide pyrethroids in two types: type I substances do not have an α-cyano group in their structure and type II compounds containing α-cyano groups in their structure [3]. Lambdacyhalothrin (LCH) is a type II pyrethroid. Poisoning with high doses of type I pyrethroids administered intravenously produces the T-syndrome consisting of aggressive sparring, sensitivity to external stimuli, tremor, and prostration in rodents. Intoxication with type II pyrethroids causes CS-syndrome, consisting of pawing and burrowing behavior, salivation, coarse tremor, progressing to choreoathetosis and clonic seizures [4]. Both types produce sympathetic activation, skin paresthesia after dermal absorption and gastrointestinal irritation in case of oral administration [5]. However, some pyrethroids have features of type I and type II [6]. Moreover, there are data that sensitivity of rodents to pyrethroids changes with age. During the neonatal period, immature rodents have limited metabolic capacity as liver cytochrome P450s and carboxylesterases that metabolize pyrethroids increase their activity during postnatal development of rats [7].

Pyrethroids act as neurotoxins via voltage gated sodium channels in neurons in insects (which are target organisms) as well as in mammals (which are non-target organisms). The voltage-sensitive sodium channels, which are the sites of insecticidal action, are also target sites in mammals. However, mammals have many sodium channel isoforms that vary in their biophysical and pharmacological properties. Pyrethroids also act on voltage-sensitive calcium and chloride channels. The peripheral-type benzodiazepine receptors enhance convulsions caused by pyrethroid actions at other target sites [8]. In the studies of pyrethroid neurotoxicity conducted by Wolansky et al., the influence of 11 pyrethroids (including cypermethrin, lambdacyhalothrin, and betacyfluthrin) administered orally on rats’ behavior was observed. The chemicals impaired motor activity in a dose-dependent manner. There was an additive effect of the mixtures [9]. Type II pyrethroids act not only via sodium channels, but also modulate chloride channels, voltage-gated calcium and potassium channels, alter the activity of glutamate and acetylcholine receptors and adenosine triphosphatases inducing DNA damage and oxidative stress in the neuronal cells. They also modulate the level of neurotransmitters: gamma-aminobutyric acid (GABA) and dopamine [10,11,12]. In humans, high doses of LCH cause excessive salivation, fatigue, coughing, and abdominal pain [13,14,15].

Humans can be exposed to LCH at work in agriculture and horticulture. It is absorbed dermally, as it is fat soluble, or by inhalation. In case of food or drink contamination, people can absorb LCH through the gastrointestinal tract. It is metabolized in the liver. The metabolites cis-3-(2-chloro-3,3,3-trifluoroprop-1-en-1-yl)-2,2-dimethylcyclopropane carboxylic acid (CFMP) and 3-phenoxybenzoic acid (3-PBA) are excreted with urine [16]. The 3-PBA is a common metabolite of pyrethroids, and it can cross the blood–brain barrier. It can be also accumulated in the brain [17]. Urinary LCH metabolite concentrations are significantly higher in rural inhabitants than in the cities [18,19]. Glutathione-S transferases (GSTs) have a function in LCH metabolism. LCH inhibits GSTs in a competitive mechanism in the livers of non-target organisms causing oxidative stress [20]. After dermal application of LCH at the dose of 0.25 mg/kg, elimination t_1/2_ is 11.2 h [16]. Traces of LCH are detected in oregano [21], prunes [22], and cabbage [23]. In Poland, large amounts of LCH are used in potato, wheat, and maize cultivation [24]. In endemic areas, capsule suspension emitting LCH 20 or 30 mg a.i./m^2^ and wettable powder (30 mg/m^2^) are used for malaria vector *An. fluviatilis* control [25]. Studies conducted in Africa, however, indicate that mosquitoes mutate fast and develop resistance to repeatedly applied insecticides [26]. LCH is used for insect control indoors at homes, in hospitals, greenhouses, to protect ornament plants, for lawn protection, as insect repellent for cattle, and for termite treatment [27]. The LCH maximum residue limit (MRL) in vegetables is 0.5 mg/kg. Djouaka et al. proved that nine days after LCH application lettuce is free from the insecticide [23]. According to manufacturer’s instruction, vegetables can be consumed 14 days after the last spraying of LCH.

Immunotoxic and immunomodulating effects were found due to exposure to many pyrethroids [15]. Cypermethrin is very immunotoxic for fish [28], and deltamethrin has immunomodulating effects in aquatic organisms [29]. In previous studies, it was shown that LCH increases tumor necrosis factor (TNF) expression in non-target organisms [30]. Therefore, a question arises whether repeated exposure of humans to LCH could affect human behavior, biochemical parameters, and blood cell counts as well as increase levels of proinflammatory cytokines.

The aim of the experiment was to study the effect of subacute poisoning with lambdacyhalothrin on locomotor activity, memory, selected biochemical parameters, tumor necrosis factor α, and interleukin 1ß in a mouse model.

## 2. Materials and Methods

The study project was accepted by The Local Ethical Committee in Lublin, Poland (permission Nr 69/2015 dated 11.12.2015). Both authors had certificates confirming training for conduction experiments on animals. The experiment was conducted according to European law regulation at the Centre for Experimental Medicine at The Medical University of Lublin. There were standard laboratory conditions (12 h light/12 h dark cycle, temperature 21–22 °C, air humidity 55–60%).

A total of 32 (16 non-gravid females and 16 males) Albino Swiss mice were used. The original source of animals was Charles River Laboratories (Cologne, Germany). The animals were bred at the Centre for Experimental Medicine at The Medical University of Lublin (breeder No 077 registered at the Ministry of Science and Higher Education, Warsaw, Poland).

The animals had free access to sterile water and animal feed (sterilized with UV) ad libitum. The feed for mice was purchased from Altromin International (Lage, Germany).

At the beginning of the experiment the mice were 6 weeks old (they were young adults). They were randomly divided into groups of 8 animals:

1. Females controls.
2. Males controls.
3. Females receiving 2 mg/kg LCH.
4. Males receiving 2 mg/kg LCH. The investigator handling the animals was blind to the treatment.

Lambdacyhalothrin was purchased from the Organic Chemistry Institute (Annopol 6, 03-236 Warsaw, Poland). It was dissolved in canola oil and administered daily by gavage. Canola oil “Kujawski”, produced by ZT “Kruszwica” S.A. was used to make the suspension. Oral LD_50_ in mice 19.9 mg/kg [27]. We administered 0.1 LD_50_ (2 mg/kg) LCH to the animals for 7 consecutive days by gavage.

Behavioral tests were performed 1 h after LCH administration as after a single oral administration of LCH at a dose of 2 mg/kg body weight the half-life of the xenobiotic is 10.27 up to 14.33 h [31].

Animals were tested in a Y-maze on day 1 and 7 in order to measure their spontaneous locomotor activity and spatial memory. Locomotor activity monitoring and fresh spatial memory in a Y-maze started 1 h after the dosing of the pyrethroid and was continued for 8 min. Spontaneous alternation in a Y-maze is a measure of fresh spatial memory. Each mouse was individually placed in the Y-maze. It consists of 3 compartments 10 cm × 10 cm × 10 cm joined at the angle of 120°. The maze has no floor. For each mouse a clean sheet of paper was placed underneath in order to prevent odor clues. Alternation (defined as consecutive entries into all 3 sections without repetitions) was scored. The percent alternation was calculated as the ratio of actual possible alternations. The ability to alternate requires that the mice remember which sections have previously been visited. The number of arm entries is also a measure of locomotor activity [32].

A passive avoidance step-through test was conducted in a two-compartment box (15 cm × 20 cm × 50 cm) with one bright and one dark compartment connected by a guillotine door. Each mouse was placed in the bright compartment and allowed to explore for 30 s, at which point the guillotine door was raised to allow the mice to enter the dark compartment. When the mouse entered the dark compartment, the door was closed, and an electrical foot shock was delivered (0.2 mA for 2 s) through the floor bars. It led to the formation of an association of the dark compartment with the punishment. The passive avoidance task combines Pavlovian contextual fear conditioning with the expression of an instrumental response. For the retention test 24 h after training, the animal was returned in the bright compartment of the apparatus for 180 s. The latency was measured on day 2 and 7. The mouse had the option to avoid or enter the dark compartment by discriminating the bright (safe) from the dark (unsafe) compartment. The rapid acquisition of not making a response indicates that the test involves learned inhibition rather than loss of an innate response tendency. Many publications point at the crucial role of the amygdala, a heavily innervated assembly of many different subnuclei that are essential for aversive learning [33,34,35,36].

On the eighth day of the experiment, the animals were weighed and then decapitated, then their venous blood, liver, and kidneys were obtained. By standard, the left kidney was collected from each mouse, and it was immediately weighed. Livers were also weighed. The weighed part of the tissue (liver, kidney) was homogenized in the proportion of 50 mg of tissue per 1 mL of lysis buffer appropriate for the protein tested from Cloud-Clone Corp (Katy, TX, USA) using an Omni Th type mechanical homogenizer (Omni International, Kennesaw, CA, USA). The prepared homogenates were centrifuged in a centrifuge (Sigma1-6P, Polygen, Edgewood, NY, USA) at 10,000× *g* for 5 min at room temperature. After centrifugation, the supernatant was carefully collected. The determinations were performed using the enzyme immunoassay method using commercial ELISA kits (Enzyme-Linked Immunoabosbent Assay) by Cloud-Clone Corp (USA): ELISA kit for Tumor Necrosis Factor Alpha (TNFα) (Cat # SEA133Mu), sensitivity 5.6 pg/mL and ELISA kit for Interleukin1 Beta (IL1b) (cat. No. SEA563Mu), sensitivity of the test 6.4 pg/mL.

Venous blood was tested at the VetDiagnostyka veterinary laboratory, Lublin, Poland. To determine peripheral blood cell counts from each animal on the last day of the experiment, 200 µL of blood was drawn for EDTA. The morphology parameters (the number of erythrocytes, leukocytes, thrombocytes, percentage of neutrophils, macrophages, eosinophils, basophils) were determined using an automatic hematological analyzer. Creatinine was measured in supernatants of kidney homogenates and alanine transaminase (ALT) activity in livers of the tested animals. Liver homogenate supernatants were diluted 10× then the result was converted to complete supernatant concentrations. An ErbaMannheim XL-60 automated biochemical analyzer was used for the determination of ALT and creatinine. A kinetic measurement method was used.

The paired Student’s t-test and Wilcoxon’s test were used to evaluate the difference between the two measurements. The Student’s t-test for independent samples and the Mann–Whitney test were used to assess the difference between the two groups. In order to evaluate statistically significant differences between consecutive measurements, the ANOVA test with repeated measures and Tukey’s test as a post hoc test were used to assess statistically significant differences between the measurements. Determining the relationship between related and unrelated variables was determined using the Pearson correlation and the Spearman correlation; *p* < 0.05 was considered statistical significance. Statistical analysis was performed with the use of Statistica v.13.0 (StatSoft) (Statsoft Sp.zo.o., Cracow, Poland).

## 3. Results

There was no statistically significant change in body weight between the control group of females and the group of females exposed to LCH (t = 2.10; *p* > 0.05). There was also no statistically significant change in body weight between the male control group and the group of males exposed to LCH (t = 0.55; *p* > 0.05) (Table 1).

There was no statistically significant difference in liver mass between the control group of females and the group of females exposed to LCH (t = −1.14; *p* > 0.05). A statistically significantly higher liver mass was recorded in the LCH groups compared to controls (t = −6.91; *p* < 0.001) (Table 2).

A statistically significantly higher kidney mass was found in the LCH group of females compared to the controls (t = −3.11; *p* < 0.01). Additionally, statistically significantly higher kidney mass was found in the male LCH group compared to the male controls (t = −2.96; *p* < 0.05) (Table 2).

There was no statistically significant difference in the white blood cell count (WBC) between the male control group and the male group exposed to LCH (t = 1.05; *p*> 0.05). However, WBC was lower in females after exposure to LCH (Table 3). There was a statistically significantly higher percentage of neutrophils in the group of LCH females as compared to the control females (t = −3.93; *p* < 0.001). There was no statistically significant difference in the percentage of neutrophils between the male control group and the group of males exposed to LCH (t = −1.22; *p* > 0.05), but the number of neutrophils in males after LCH was higher than in the control group (Table 3). There was no statistically significant difference in the percentage of lymphocytes, monocytes, eosinophils, basophils, nor platelets between the control group of females and the group of females exposed to LCH. Likewise, it was in the groups of males (Table 3). The red blood cell count (RBC) was statistically significantly lower in the groups exposed to LCH compared to controls (females (t = 2.22; *p* < 0.05, males t = 9.74; *p* < 0.001) (Table 3).

There was no statistically significant difference in TNFα levels in the kidneys and livers between control and LCH groups (Table 4). A statistically significant elevation of Il 1ß was demonstrated in the livers and kidneys in the male LCH group compared to the control group (t = −9.30; *p* < 0.01) (Table 4.).

ALT activities in mice livers are shown in Table 5. There were no statistically significant differences among the groups. A statistically significantly higher level of creatinine was found in the group of females after LCH compared to the control group (t = 3.35; *p* < 0.01) (Table 5). A similarly statistically significant difference was between males after LCH compared to controls (t = 3.42; *p* < 0.01).

There was no statistically significant influence of LCH on memory retention in passive avoidance in males nor females on day 2, but males had better memory retention than females. On day 7, memory retention was significantly impaired in males (Table 6). A significant correlation (*p* = 0.018513) was found between creatinine and Il1ß concentrations in mice kidneys.

There were no statistically significant differences in locomotor activity among the groups on day 1 nor on day 7 an in the % of logical alternations on day 1 and day 7 (Table 7).

## 4. Discussion

In our present study the 7-day exposure to LCH did not affect memory nor locomotor activity on day 1 and day 2 in mice. Memory retention was tested in passive avoidance task on day 2 and day 7. Only in males after 7-day exposure to LCH, there was a decrease in memory retention possibly due to repeated exposure to the xenobiotic. In the classic experiment conducted by Crofton and Reiter in 1984 on rats exposed to deltamethrin at the dose of 2, 6, or 8 mg/kg or cismethrin at the dose of 6, 12, 18, or 24 mg/kg administered orally in corn oil, both pyrethroids produced a similar dosage-dependent decrease in motor activity without cumulative effects [37]. LCH significantly reduces locomotor activity in target organisms and non-target insects [38] and after high dose exposure of mammals [9]. It strongly affects fish and crustaceans such as daphnia swimming behavior [39]. In our earlier experiments, the effect of LCH on motor activity and memory processes in mice previously exposed to transient ischemia of the brain by temporary ligation of the common carotid arteries was investigated. LCH in the form of a commercial preparation (Karate 025 EC) was administered to mice in such dilution that the animals received 0.1 LD_50_ intraperitoneally (LD_50_ = 6.9 mg/kg bw ip). Their motor activity, motor coordination, fresh spatial memory, and memory retention were tested. It was found that exposure to LCH and prior cerebral ischemia significantly decreased the animal’s motor activity. No significant memory impairment was observed [32]. The differences between the former and the present study result from different dosing and other routes of administration. This time we wanted to mimic possible human exposure to LCH via food and contaminated drinks. Intraperitoneal injections are unlikely in real life.

Fish and crustaceans are the most sensitive to pyrethroids apart from insects. Birds and mammals have constant internal body temperature, which is higher than in insects therefore they show less effects of LCH intoxication. Mammals have a higher rate of pyrethroid metabolism than insects, and their sodium channels are less sensitive to pyrethroids’ toxicity than in the target organism [1]. However, neonatal exposure to permethrin may cause permanent memory deficits in later life in rats [40].

Our results are similar to ones obtained by other teams [11,41]. In the experiment of Aouey et al. in male rats, LCH was administered i.p. at a dose of 6.2 or 31.1 mg/kg for 60 days. Liver LCH metabolites, asparagine transaminase (ASAT), ALAT, and lactate dehydrogenase (LDH) were assessed; lipid peroxidase (which is a marker of oxidative stress), the content of antioxidants: vitamin C, thiols, antioxidant enzymes’ activity: catalase activity (CAT), superoxide dismutase activity (SOD), glutathione peroxidase activity (GPx), and proinflammatory TNFα were estimated. Accumulation of LCH metabolites was demonstrated in the liver in a manner proportional to the dose of LCH. Moreover, a dose-proportionate statistically significant increase in the activity of hepatic oxidative markers was recorded. The activities of ASAT, LDH, and ALAT increased. The level of reactive oxygen species, depending on the administered dose of LCH, was statistically significantly increased. TNFα levels were significantly increased in each group of animals exposed to LCH. There was a decrease in the level of non-enzymatic antioxidants and a decrease in the activity of SOD, CAT, and GPx in the groups exposed to LCH. Enhanced transcription of TNFα and IL-1β after LCH was established [11]. The cited study focused on hepatotoxic effects of LCH. We, however, also aimed to detect nephrotoxic effects of exposure to the pyrethroid. Our study showed that not only livers but also kidneys are damaged due to subacute poisoning with LCH. It is due to the fact that kidneys are engaged in elimination of its metabolites.

The study of Parwar et al., similar to ours, was carried out on mice. LCH was administered to the animals orally at doses of 0.5 mg/kg, 1 g/kg bw 2 mg/kg bw for 29 days. Blood parameters of oxidative stress and enzymatic antioxidants were analyzed. The studies showed that all serum parameters tested differ statistically significantly in a dose-dependent manner compared to the control group. A statistically significant increase in the concentration of malondialdehyde in the kidneys was found after exposure to higher doses of LCH. There was a significant decrease in the levels of thiols in the kidneys in all LCH groups. Medium and high doses of LCH caused a significant decrease in SOD and catalase activity in the kidneys. Histopathological evaluation of the kidneys revealed hemorrhages in the cortex and core, tubular degenerative changes with closure of the lumen and reduction in Bowman’s capsule space [41]. In another study, a positive correlation was observed between elevated concentrations of pyrethroid metabolites in the urine of men, and the occurrence of sperm abnormalities decreased testosterone levels and fertility disorders [42].].

According to Gargouri et al. rats exposed bifenthrin for 60 days exhibited spatial and cognitive impairments and memory dysfunction. TNFα and interleukin1ß expression increased. This was accompanied by oxidative stress in the hippocampus of treated animals. The authors conclude that exposure to bifenthrin induces neuronal damage, oxidative stress, and neuroinflammation in the hippocampus, which might lead to cognitive and memory impairment [43]. Dar et al. administered bifenthrin to rats for up to 30 days and produced the highest oxidative stress in the liver followed by the kidney and lung [44]. The in vitro study conducted by Bordoni et al. showed that permethrin also produces pro-oxidant activity in a dopaminergic cell line [45]. Anitha et al. found out that exposure to deltamethrin increases the levels of serotonin, dopamine, and noradrenaline in rats. Interestingly, their study suggests that deltamethrin in combination with other insect repellents show an antagonistic effect against oxidative stress [46].

In the study of Mohi El-Din et al., LCH was administered to rats at the dose of 9.34 mg/kg ip. The authors analyzed blood morphology changes in the course of subacute poisoning, changes in the lungs, and the beneficial effects of ginseng at a dose of 200 mg/kg as well as good effects of garlic at a dose of 100 mg/kg [47]. That was due to high levels of natural antioxidants in ginseng and garlic.

In our study, kidney enlargement, creatinine, and IL1ß increase in the kidneys confirm nephrotoxic effect of LCH. Fetoui et al. demonstrated in a study on rats exposed to LCH that the administration of vitamin C improves the parameters of oxidative stress and biochemical parameters. Creatinine urea and uric acid levels are falling in the LCH and vitamin C groups [48].

There are many reports confirming that exposure on pyrethroids causes oxidative stress and in this mechanism causes an increase in the expression of genes encoding proinflammatory cytokines [49,50,51,52,53,54,55]. The interleukin1β is primarily synthesized in macrophages and monocytes. It is secreted into the blood, thanks to which it has a systemic effect and is one of the first cytokines to appear during inflammation [49]. TNFα is also produced primarily by macrophages and monocytes. TNFα activates monocytes, neutrophils, and macrophages. Moreover, it induces the release of cytokines (interferon-γ, interferon-β, IL-1, IL-6, granulocyte-colony stimulating factor, monocyte colony-stimulating factor, platelet-derived growth factor, platelet-activating factor), prostaglandins, and leukotrienes. So, there is a TNFα increase, and later, there is an Il1ß peak. Interleukins are also involved in hematopoietic and pro-inflammatory processes and anti-inflammatory as signaling molecules [56]. This is why there were changes in white and red blood cell counts due to LCH poisoning in our study.

## 5. Conclusions

Subacute poisoning with a low dose of LCH does not significantly affect memory or locomotor activity but increases proinflammatory interleukin 1ß in male livers and kidneys and reduces the white and red blood cell counts.

## Figures and Tables

**Table 1:** Body mass changes in the animals.

Group	Day 1 [g]	Day 2 [g]	Day 3 [g]	Day 4 [g]	Day 5 [g]	Day 6 [g]	Day 7 [g]
Control females (mean ± SD)	20.53 ± 0.84	20.61 ± 1.14	20.96 ± 1.40	21.33 ± 1.58	21.38 ± 1.52	21.79 ± 1.57	22.53 ± 1.52
LCH females (mean ± SD)	23.88 ± 0.83	23.13 ± 0.64	23.88 ± 0.64	24.25 ± 0.71	24.25 ± 0.89	24.13 ± 0.64	24.25 ± 0.89
Control males (mean ± SD)	24.41 ± 2.85	24.95 ± 3.66	25.36 ± 3.74	26.15 ± 3.95	26.56 ± 3.92	26.96 ± 4.01	27.21 ± 4.18
LCH males (mean ± SD)	30.75 ± 2.82	31.50 ± 2.83	33.25 ± 3.49	32.25 ± 2.60	32.00 ± 2.77	32.50 ± 2.78	32.50 ± 2.93

LCH: Lambdacyhalothrin.

**Table 2:** Liver and kidney mass in the animals.

Group	Kidney Mass [g]	Liver Mass [g]
Control females (mean ± SD)	0.16 ± 0.02	1.24 ± 0.18
LCH females (mean ± SD)	0.18 ± 0.01 *	1.33 ± 0.16
Control males (mean ± SD)	0.16 ± 0.02	1.33 ± 0.21
LCH males (mean ± SD)	0.18 ± 0.01 *	2.04 ± 0.20 *

* *p* < 0.05 vs. controls.

**Table 3:** Blood morphology in the animals.

Group	WBC [×1000/µL]	Neutrophils [%]	Lymphocytes [%]	Monocytes [%]	Eosinophils [%]	Basophils [%]	Erythrocytes [×10^6^/µL]	Platelets [×1000/µL]
Control females (mean ± SD)	8.04 ± 2.72	10.20 ± 2.54	82.60 ± 8.92	2.48 ± 7.97	0.050 ± 0.107	0.68 ± 0.38	10.63 ± 0.52	853.88 ± 188.24
LCH females (mean ± SD)	3.07 ± 1.25 *	16.63 ± 3.87 *	82.10 ± 3.65	2.89 ± 0.7	0.075 ± 0.149	0.31 ± 0.11	9.01 ± 1.99 *	968.63 ± 93.23
Control males (mean ± SD)	7.44 ± 4.01	13.13 ± 6.61	83.98 ± 6.97	2.60 ± 1.19	0.001 ± 0.001	0.30 ± 0.11	10.88 ± 0.64	1083.88 ± 204.30
LCH males (mean ± SD)	5.83 ± 1.71	16.18 ± 2.55	82.00 ± 3.47	1.88 ± 0.4	0.025 ± 0.071	0.16 ± 0.11	7.29 ± 0.82 *	993.50 ± 117.98

* *p* < 0.05 vs. controls, WBC: white blood cell.

**Table 4:** TNFα and IL1ß in the livers and kidneys.

Group	TNFα in the Kidney [pg/mL]	TNFα in the Liver [pg/mL]	Il1ß in the Kidney [pg/mL]	Il1ß in the Liver [pg/mL]
Control females (mean ± SD)	16,239.80 ± 4356.58	29,974.61 ± 5103.88	13,974.74 ± 1768.01	22,977.01 ± 5215.69.
LCH females (mean ± SD)	21,769.34 ± 6776.57	29,427.43 ± 6507.89	16,991.41 ± 3845.08	25,196.31 ± 13,040.87
Control males (mean ± SD)	18,129.68 ± 647.14	3,140,680 ± 7669.35	14,010.55 ± 1708.89	22,266.82 ± 5594.18
LCH males (mean ± SD)	26,465.60 ± 11,594.85	34,619.05 ± 10,617.62	27,392.20 ± 3695.44 *	37,343.97 ± 37,343.97 *

* *p* < 0.05 vs. controls, TNF: tumor necrosis factor.

**Table 5:** Alanine transaminase (ALT) activity and creatinine concentration.

Group	ALT [U]	Creatinine [g]
Control females (mean ± SD)	7537.50 ± 1890.80	0.11 ± 0.01
LCH females (mean ± SD)	7453.75 ± 1012.35	0.15 ± 0.01 *
Control males (mean ± SD)	8155.00 ± 1149.85	0.12 ± 0.01
LCH males (mean ± SD)	8572.50 ± 559.79	0.15 ± 0.03 *

* *p* < 0.05 vs. controls.

**Table 6:** Results of passive avoidance task on day 2 and 7.

Group	Memory Retention Day 2 [Median (Q1; Q3)]	Memory Retention Day 7 [Median (Q1; Q3)]
Control females	134 (77; 180)	118 (80;180)
LCH females	105 (18; 180.00)	97 (10;180)
Control males	180 (123; 180)	180 (180;180)
LCH males	180 (149; 180)	98 * (18;180)

* *p* < 0.05 vs. controls.

**Table 7:** Locomotor activity and fresh spatial memory in Y-maze on day 1 and day 7.

Group	Locomotor Activity on Day 1 (Number of Arm Entries)	Locomotor Activity on Day 7 (Number of Arm Entries)	% of Logical Alternations in the Y-Maze on Day 1	% of Logical Alternations in the Y-Maze on Day 7
Control females (mean ± SD)	46 ± 9	33 ± 4	54 ± 2	64 ± 2
LCH females (mean ± SD)	51 ± 4	37 ± 1	67 ± 8	51 ± 6
Control males (mean ± SD)	33 ± 3	29 ± 3	60 ± 5	54 ± 2
LCH males (mean ± SD)	35 ± 9	35 ± 3	60 ± 5	59 ± 9
